# Is smoking associated with increased prescription opioid use and misuse? Evidence from U.S. adults

**DOI:** 10.1097/JS9.0000000000000917

**Published:** 2023-11-22

**Authors:** Kun Han, Tianhong Wang, Feng Shen, Tao Li, Leng Zhou

**Affiliations:** a Department of Anesthesiology, West China Second Hospital of Sichuan University; b Key Laboratory of Birth Defects and Related Diseases of Women and Children, Sichuan University, Ministry of Education; c Department of Anesthesiology, West China Hospital of Sichuan University; d Department of Anesthesiology, Laboratory of Mitochondria and Metabolism, National Clinical Research Center for Geriatrics, West China Hospital, Sichuan University, Chengdu, Sichuan Province, People’s Republic of China

*Dear Editor*,

Globally, over one billion people smoke, accounting for nearly 20% of the population^1^. Smoking is associated with an increased risk of diseases like cancer and cardiovascular disease and with premature death and socioeconomic burden^2^. As proposed in a recent article published in the *International Journal of Surgery*, preoperative smoking cessation interventions are cost-effective for preventing surgical complications^3^. Substantial evidence also links smoking with chronic pain via mechanisms including inflammation, neural damage, and altered pain regulation^4^. Furthermore, while opioids are widely used for chronic pain, their long-term use can lead to tolerance, dependence, and misuse^5^. Given the pharmacological parallels between nicotine and opioids, investigating if smoking predisposes people to opioid misuse is warranted. This study aims to explore associations between smoking, opioid use/duration, and smoking cessation’s impact on U.S. adults’ opioid use. We hope this finding will provide education and guidance for interventions targeting the public health impacts of smoking and opioid misuse.

Data were extracted from the National Health and Nutrition Examination Survey (NHANES; 2015–2016; *n*=9544), a nationally representative survey of health and nutrition in the U.S. Participants with incomplete smoking questionnaire data or missing information on prescription medication were excluded from the analysis. Additionally, participants with missing covariates, such as basic sociodemographic information, hypertension, and diabetes complications, were also excluded. As a result, 5381 individuals were included in the final analysis (Supplementary Figure 1, Supplemental Digital Content 1, http://links.lww.com/JS9/B338). Participants self-reported smoking status, prescription medication uses in the past 30 days, and duration of medication use. Prescription opioid use was defined as the reported use of specified opioid analgesics. Covariates included sociodemographics, body mass index, hypertension, and diabetes. We used logistic regression to assess associations between smoking status and prescription opioid use, and multinomial logistic regression for associations with opioid use duration (<90 vs. ≥90 days), adjusting for covariates. NHANES was approved by the National Center for Health Statistics Ethics Review Board, and all participants provided written consent.

In this U.S. nationally representative sample of 5381 adults (median age 49 years; 52% female), 6.9% reported past-month prescription opioid use, and 5.5% reported use for ≥90 days (Table 1). Overall, 41.8% were current/former smokers. Smokers tended to be older, have higher body mass index (former smokers), lower education, and were more often unmarried than non-smokers. Diabetes and hypertension were more prevalent in former versus non-smokers. In unadjusted models, former [odds ratio (OR) 1.94, 95% confidence interval (CI) 1.50–2.49] and current smokers (OR 2.30, 95% CI 1.77–2.98) had higher odds of prescription opioid use compared to non-smokers (Fig. 1A). In adjusted analyses, former and current smokers had 1.67 (95% CI 1.27–2.19) and 1.60 (95% CI 1.40–1.83) times higher odds of prescription opioid use versus non-smokers, respectively (Fig. 1B). The association between smoking status and prescription opioid use was more pronounced for long-term versus short-term use. Current smokers had higher odds of long-term opioid use than non-smokers (OR 2.47, 95% CI 1.85–3.31). Similarly, former smokers had higher odds of long-term use (OR 2.25, 95% CI 1.70–2.98). However, there was no significant difference in short-term opioid use between former smokers and non-smokers (OR 1.60, 95% CI 0.60–1.90, *P*=0.79) (Fig. 1C).

**Table 1:** Demographic characteristics stratified by exposure.

	Total	Never	Ever	Now	*P*
Number of patients	5381	3132	1237	1012	
Age, years, mean (SD)	49.00 (34.00, 63.00)	45.00 (32.00, 61.00)	60.00 (44.00, 71.00)	46.00 (33.00, 59.00)	<0.001
Age, years, *n* (%)					<0.001
20–39	1837 (34.1)	1208 (38.6)	246 (19.9)	383 (37.8)	
40–59	1786 (33.2)	1053 (33.6)	352 (28.5)	381 (37.6)	
60–79	1428 (26.5)	692 (22.1)	501 (40.5)	235 (23.2)	
>80	330 (6.1)	179 (5.7)	138 (11.2)	13 (1.3)	
Sex, *n* (%)					<0.001
Male	2567 (47.7)	1203 (38.4)	767 (62.0)	597 (59.0)	
Female	2814 (52.3)	1929 (61.6)	470 (38.0)	415 (41.0)	
BMI, kg/m^2^, mean (SD)	28.40 (24.50, 33.20)	28.40 (24.30, 33.20)	29.10 (25.50, 33.40)	27.70 (23.98, 33.00)	<0.001
BMI, kg/m^2^, *n* (%)					<0.001
<20	223 (4.1)	128 (4.1)	29 (2.3)	66 (6.5)	
20–24.9	1260 (23.4)	777 (24.8)	232 (18.8)	251 (24.8)	
25–29.9	1724 (32.0)	973 (31.1)	431 (34.8)	320 (31.6)	
>30	2174 (40.4)	1254 (40.0)	545 (44.1)	375 (37.1)	
Race, *n* (%)					<0.001
Hispanic	1647 (30.6)	1041 (33.2)	369 (29.8)	237 (23.4)	
Non-Hispanic Black	1134 (21.1)	649 (20.7)	203 (16.4)	282 (27.9)	
Non-Hispanic White	1760 (32.7)	854 (27.3)	539 (43.6)	367 (36.3)	
Non-Hispanic other	840 (15.6)	588 (18.8)	126 (10.2)	126 (12.5)	
Education, *n* (%)					<0.001
Less than high school	1268 (23.6)	695 (22.2)	288 (23.3)	285 (28.2)	
High school or equivalent	1168 (21.7)	588 (18.8)	289 (23.4)	291 (28.8)	
Some college	1595 (29.6)	873 (27.9)	406 (32.8)	316 (31.2)	
College or above	1350 (25.1)	976 (31.2)	254 (20.5)	120 (11.9)	
Marital, *n* (%)					<0.001
Married/living with partner	3269 (60.8)	1969 (62.9)	788 (63.7)	512 (50.6)	
Never married	984 (18.3)	601 (19.2)	140 (11.3)	243 (24.0)	
Widowed/Divorced/Separated	1128 (21.0)	562 (17.9)	309 (25.0)	257 (25.4)	
Diabetes mellitus, *n* (%)	909 (16.9)	473 (15.1)	298 (24.1)	138 (13.6)	<0.001
Hypertension, *n* (%)	1945 (36.1)	999 (31.9)	554 (44.8)	392 (38.7)	<0.001
Length of drug use, *n* (%)					<0.001
Never	5009 (93.1)	2979 (95.1)	1125 (90.9)	905 (89.4)	
Short term	78 (1.4)	40 (1.3)	16 (1.3)	22 (2.2)	
Long term	294 (5.5)	113 (3.6)	96 (7.8)	85 (8.4)	

Data are present in *n* (%) or mean (SD).

BMI, body mass index; SD, standard deviation.

**Figure 1:**
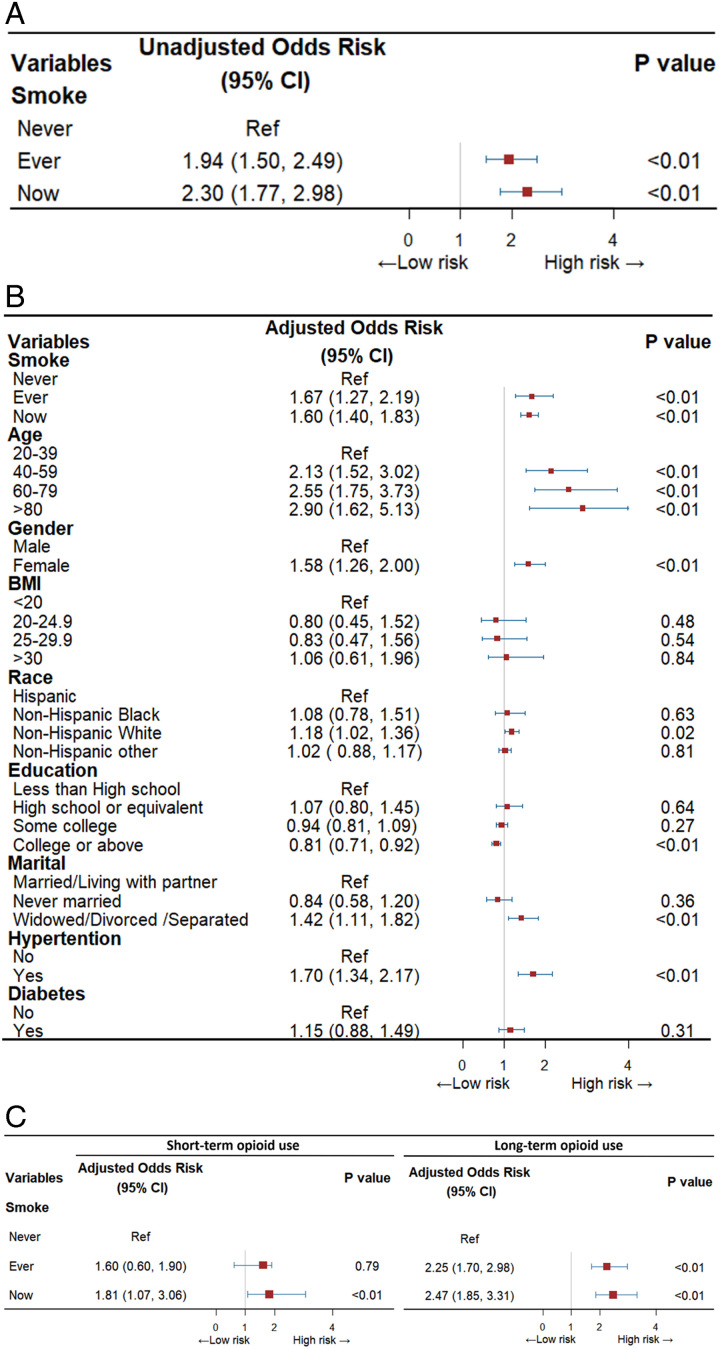
(A) Forest plot of the relationship between smoking and opioid use (unadjusted models). (B) Forest plot of the relationship between smoking and opioid use (adjusted models). (C) Forest plot of former or current smoking versus short-term or long–term opioid use.

In conclusion, this study found significant associations between smoking and prescription opioid use, especially long-term use, in a nationally representative U.S. sample. The shared pharmacological effects, psychological factors, and social influences linking smoking and opioid misuse likely underpin these associations. Comprehensive public health strategies encompassing education, regulation, and support services are warranted to address the intersection of smoking and opioid misuse and mitigate the substantial individual and societal harms. Further research should explore longitudinal impacts and refine understanding of mechanisms influencing smoking and opioid co-use.

## Ethical approval

Not applicable.

## Sources of funding

This work was supported by the key research and development program of Sichuan Province (2023YFS0295).

## Authors contribution

K.H. and T.W.: conceptualization, data acquisition, and writing; K.H. and F.S.: statistical analysis, creation of figure, and writing; L.Z. and T.L.: review, editing, and supervision.

## Conflicts of interest disclosure

The authors declare no conflicts of interest.

## Research registration unique identifying number (UIN)

1. Name of the registry: not applicable.
2. Unique identifying number or registration ID: not applicable.
3. Hyperlink to your specific registration (must be publicly accessible and will be checked): not applicable.

## Guarantor

All authors.

## Data availability statement

Data are available from the corresponding author if justification for the requirement is justified.

## Supplementary Material
