# Supplementary figures and images for: Is smoking associated with increased prescription opioid use and misuse? Evidence from U.S. adults
Source: Int J Surg. 2023 Nov 22;110(2):1310–2. doi: 10.1097/JS9.0000000000000917 (PMC10871563; doi:10.1097/JS9.0000000000000917)

**
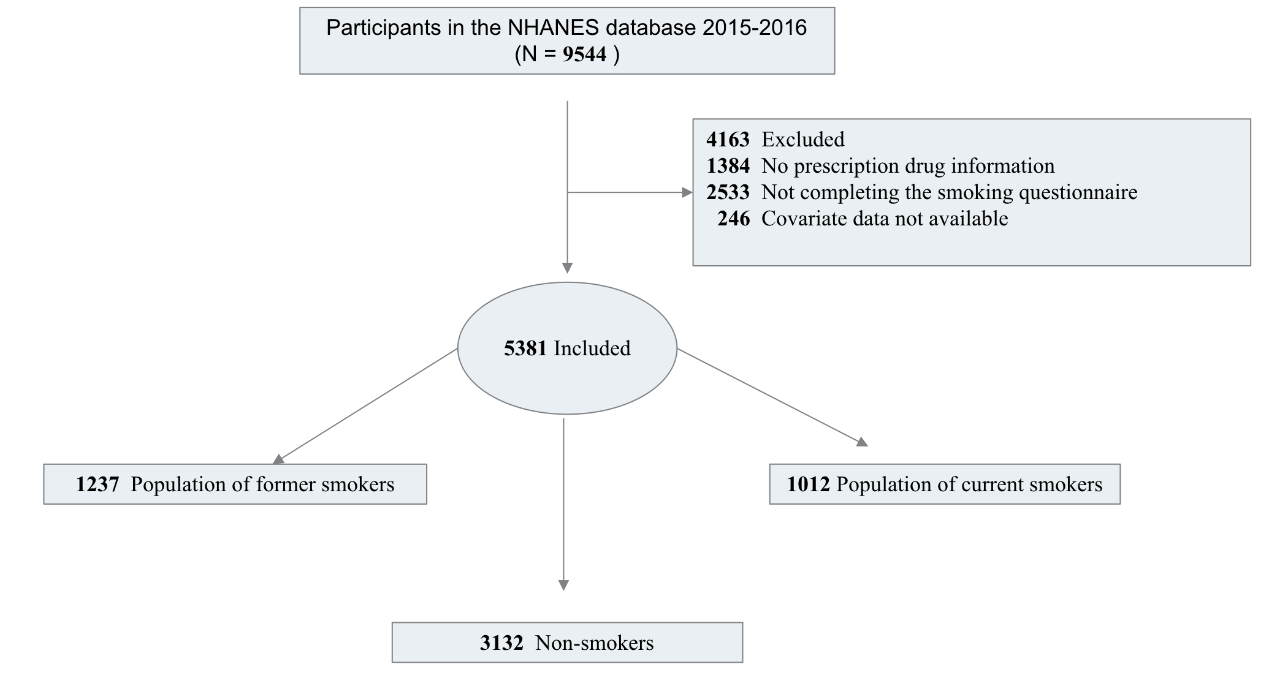
**

**Supplementary Figure 1** Flowchart of inclusion and exclusion.

Supplement: Supplementary file 1 [file js9-110-1310-s001.docx]
